# MALDI-TOF MS Detection of Oxidized Major Phospholipids in Biological Samples Using Conventional Matrices and 1‑Pyrenebutyric Hydrazide

**DOI:** 10.1021/jasms.5c00196

**Published:** 2025-09-16

**Authors:** Patricia Prabutzki, Jürgen Schiller, Kathrin M. Engel

**Affiliations:** Institute of Medical Physics and Biophysics, Faculty of Medicine, 9180 Leipzig University, Härtelstrasse 16-18, 04107 Leipzig, Germany

## Abstract

Oxidized lipids are involved in many widespread diseases associated with dysregulated lipid metabolism and/or low-level chronic inflammation. An increase in reactive oxygen species due to redox imbalance leads to the generation of various lipid peroxidation products, including lysolipids and truncated carbonyl compounds, particularly carboxylic acids and aldehydes. The latter can readily react with other biomolecules, such as DNA or proteins, and thereby impair their biological functions. Despite the growing interest in the role and function of oxidized lipids, their analysis remains challenging. This is due to several factors affecting their straightforward analysis, including their low abundance, their structural diversity, and their transient nature as well as method-specific factors such as the impact of matrix-assisted laser desorption/ionization (MALDI) matrices on the detectability of such oxidized lipids. Here, we evaluate the detectability of different oxidized major phospholipids, using different MALDI matrices including 2,5-dihydroxybenzoic acid, 4-(dimethylamino)­cinnamic acid, and 9-aminoacridine with rat liver extracts serving as a proxy for complex biological specimen. We also show that 1-pyrenebutyric hydrazide is a suitable matrix compound for the MALDI mass spectrometric analysis of native and oxidized phosphatidylcholine and phosphatidyl­ethanolamine lipids, as it can function both as a derivatization agent for truncated oxidized aldehyde lipids and as a regular (UV)-MALDI matrix.

## Graphical abstract

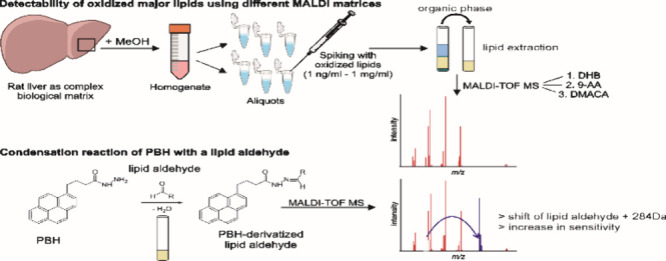

## Introduction

Cell membranes are built from proteins, cholesterol, and particularly phospholipids (PL) with phosphatidylcholine (PC) being one of the major amphiphilic constituents.^1,2^ PC are also the main PL of low-density lipoprotein particles and lipid droplets.^3^ Comprised of two fatty acyl residues, some PC molecules contain at least one polyunsaturated acyl residue, which is highly prone to lipid peroxidation by the attack of free radicals or reactive oxygen species (ROS) in general.^4^ The oxidation of PL *in vivo* occurs by ROS, which are, for example, produced by immune cells under inflammatory conditions.^5^ The free radical-induced oxidation of unsaturated PC, such as PC 16:0/18:1 (POPC) and PC 16:0/18:2 (PLPC), leads to peroxidized compounds (hydroperoxides) as primary products, which subsequently decay under generation of aldehydes and carboxylic acids as secondary oxidation products; i.e., scission occurs at the positions of the original double bonds. Hence, lipid peroxidation often results in highly reactive PC-containing oxidized PL (oxPL) capable of initiating a radical chain reaction. The higher the double bond number of one fatty acyl residue, the higher is the peroxidation rate, which (in combination with reduced diffusion pathways) led to the assumption that intramolecular peroxidation events are much faster than intermolecular ones.^6^ Thus, oxPL are primarily nonenzymatic products. Oxidized lipids were shown to be sensitively detectable using electrospray ionization mass spectrometry with detection limits in the nM range.^7,8^ Selected oxPL and LPC show biological activities similar to the platelet-activating factor and are capable of destabilizing biological membranes.^9^ Apart from their presence in cellular membranes, oxPL are transported via lipoprotein a (Lp­(a)), which is synthesized in the liver.^10^ OxPL promote inflammatory and proatherogenic processes, participate in apoptotic processes, and contribute to the progression of cardiovascular disease, e.g., myocardial infarction, stroke, and calcific aortic valve stenosis. The levels of Lp­(a) and oxPL-apoB were found to positively correlate with stenosis progression, especially in younger patients.^11^ In hepatocytes, oxidative stress can lead to liver steatosis and subsequently to nonalcoholic fatty liver disease (NAFLD) and eventually steatohepatitis (NASH).^12^ The accumulation of oxPL in NASH was obvious from a study that found higher levels of oxPL in human liver sections with a fibrosis score of ≥1 compared to healthy livers.^13^ An immunohistochemical approach showed that oxPC localized mainly in steatotic and ballooned hepatocytes as well as macrophages in NASH.^14^ However, it is a serious disadvantage of this approach that no further differentiation of the oxidation products can be made. Since the prevalence of NASH is continuously increasing and approximately 1/3 of the global population already meets the criteria for NAFLD, making it the most abundant chronic liver disease,^15^ reliable detection of oxPL in tissues of medical relevance by matrix-assisted laser desorption/ionization mass spectrometry (MALDI MS) might provide important insights into ongoing pathological events.^16^

PL are degraded to lysophospholipids (LPL) either under physiological (e.g., the acrosome reaction in sperm cells which is accompanied by phospholipase A_2_ (PLA_2_) activity) or pathophysiological circumstances (e.g., ROS generated under inflammatory conditions).^5^ Within healthy cells, LPL can be recycled to PL to maintain cellular homeostasis.^17,18^ However, this mechanism is impaired in different diseases such as liver steatosis. The problem with LPL is their detergent-like character; i.e., they are able to harm membrane integrity. Among LPL, lysophosphatidylcholine (LPC) is the most abundant LPL in mammals with a concentration of approximately 100–500 μM in healthy human blood plasma (cf. lysophosphatidyl­ethanolamine (LPE), 5–20 μM; lysophosphatidylserine, 0.1 μM).^19^

The quantitative analysis of (low abundant) LPL in tissues is less straightforward than in plasma, as there are so far no established reference values. In approaches that investigate the lipid composition of tissues from tissue homogenates, differentiation between intra- and extracellularly generated LPL is not possible. The same is true for complex oxPL, which is typically detected from liquid tissue extracts by semitargeted liquid chromatography–tandem mass spectrometry (LC–MS/MS) analysis.^20^ Thus, no conclusions can be drawn about their origin or prior localization. This issue can be presumably addressed by high-resolution mass spectrometry imaging (MSI) in the near future.

Because of the permanent positive charge of their choline headgroup, PC and its oxidation products such as LPC and 1-hexadecanoyl-2-(9-oxo-nonanyl)-*sn*-glycero-3-phosphocholine (abbreviated as PON-PC or PC 25:1;O, systematically named PC 16:0/9:0<oxo> ^21^) are very sensitively detectable by MS approaches in the positive ion mode, even in lipid mixtures.^22^ For example, MALDI MS allows for the detection of lipids in submicroliter quantities if a suitable matrix substance is chosen.^23^ However, the detection of endogenous oxPL in biological material is typically aggravated by their very low abundance in comparison to their nonoxidized counterparts and their transient nature. Tailored MALDI protocols using, e.g., ZrO_2_ nanoparticles in combination with 4-aminobenzoic acid as derivatization agent allowed for the sensitive detection of oxPL with truncated aldehydic residues via nanoparticle capturing.^24^ However, this approach required both prior enrichment using micropreparative solid phase extraction and nanoparticle-binding as well as prior derivatization in solution. Since nanoparticles are often not commercially available, their application is limited to laboratories that have the required synthetic and analytical capabilities. Therefore, it would be advantageous if all required components would be commercially available. 1-Pyrenebutyric hydrazide (PBH) is such a commercially available agent, which is commonly used as a fluorescence derivatization reagent for the detection of carbonyl compounds and has prior been used for the derivatization-based analysis of oxPL by HPLC-MS/MS.^25^ PBH was also shown to increase the sensitivity for the detection of oligosaccharides using MALDI MS.^26^ While different reactive matrices possessing ionization-enhancing aromatic groups have already been applied in MALDI MS for on-target and on-tissue derivatization before,^27,28,24^ PBH has so far not been used for the MALDI-based analysis of lipids or their oxidation products.

In the present study, we investigated the detection limits of the artificially introduced oxPL species LPC 17:0 and PC 16:0/9:0<oxo>, which commonly are not detectable in rat liver tissue homogenates by MALDI MS, even if different MALDI matrices are utilized. Furthermore, we show that PBH as a derivatization agent significantly enhances the detectability of PC- and phosphatidylethanolamine (PE)-derived aldehydes. This would be beneficial for MSI studies aiming for the spatially resolved investigation of aldehyde-containing oxPL in tissues. However, it must be noted that matrix deposition itself can impair derivatization efficiency,^29^ and thus, the applicability of PBH for MSI needs to be evaluated and optimized in the future. This is not the subject of this paper.

## Experimental Section

### Chemicals

All substances were purchased in the highest commercially available purity. The MALDI matrices 2,5-dihydroxybenzoic acid (DHB), 4-(dimethylamino)­cinnamic acid (DMACA), 9-aminoacridine (9-AA), and PBH as well as copper (II) sulfate pentahydrate and ammonium bicarbonate (ABC) were obtained from Sigma-Aldrich Chemie GmbH (Taufkirchen, Germany). l-Ascorbic acid sodium salt was obtained from Fluka (Buchs, Switzerland). Chloroform was obtained from Merck KGaA (Darmstadt, Germany). Methanol, 2-propanol, and water were purchased from Biosolve BV (Valkenswaard, The Netherlands). Standard lipid substances were obtained from Avanti Polar Lipids (Birmingham, AL, USA).

### Lipid Extraction from Rat Livers

Rat liver was used as a complex biological matrix. Livers were dissected from two female adult Sprague-Dawley rats (no. 20695, born 3/11/2024 and, no. 21850, 7/24/2024, TVV 32/23) and served as biological replicates. Livers (approximately 4.5 g) were homogenized on ice in 10 mL of methanol each using an ultra turrax (IKA-Werke GmbH & Co. KG, Staufen, Germany).

### Dilution of Standard Lipids

LPC 17:0 (Avanti, no. 855676) was dissolved in methanol to get a stock solution of 10 mg/mL. For PC 16:0/9:0<oxo> (no. 870605) and SM 18:1;O2/16:1 (no. 860684), a stock solution of 1 and 2 mg/mL were prepared in methanol, respectively. Lipid stock solutions were added to 100 μL of liver homogenate each and mixed thoroughly to obtain the following final concentrations of the lipid standards: 1 mg/mL, 0.5 mg/mL, 0.1 mg/mL, 50 μg/mL, 10 μg/mL, 5 μg/mL, 1 μg/mL, 0.5 μg/mL, 0.1 μg/mL, 50 ng/mL, 10 ng/mL, 5 ng/mL and 1 ng/mL. Subsequently, homogenates were transferred to glass vials and lipids were extracted by adding 1 mL of methanol, 1.25 mL of chloroform and 1 mL of water and vigorous mixing for 30 s. Phase separation was achieved by centrifugation at 3500 rpm for 5 min. The lower, organic phase was transferred to a new glass vial and extraction was repeated by adding 1 mL of chloroform, vigorous mixing and centrifugation. Organic extracts were combined, evaporated to dryness, and redissolved in 100 μL of chloroform/methanol (1:1, *v/v*).

### In Vitro Oxidation of PE 18:0/20:4

*In vitro* oxidation of PE 18:0/20:4 was performed based on Lange et al.^30^ with slight adaptations. A volume of 500 μL of 3 mmol/L ABC buffer was added to 10 mg of dried PE 18:0/20:4 and sonicated for 45 min. After the addition of 250 μL of 1.2 mmol/L CuSO_4_·5H_2_O (final concentration: 0.3 mmol/L) and 250 μL of 2.4 mmol/L Na-l-ascorbate (final concentration: 0.6 mmol/L) the solution was incubated for 24 h under continuous shaking at 37 °C. Lipids were extracted by adding chloroform/methanol (1:1, *v/v*) to the solution and mixed for 30 s. A complete phase separation was achieved after centrifugation for 10 min at 4000 rpm. The lower chloroform phase was isolated and dried in a vacuum concentrator.

### Matrix-Assisted Laser Desorption/Ionization Time-of-Flight Mass Spectrometry (MALDI-TOF MS)

Different matrices were used for MALDI-TOF MS analysis of the lipid extracts to test the achievable sensitivity to added (oxidized) lipids in rat livers. DHB was dissolved in methanol to a final concentration of 0.5 mol/L.^31^ 10 mg of 9-AA was dissolved in 1 mL of acetonitrile/2-propanol (4:6, *v/v*).^32^ The performance of DMACA as matrix substance was tested by mixing DMACA with either tetrahydrofuran (THF)^33^ or acetonitrile/THF (1:3, *v/v*) to get clear solutions of 5, 10, and 20 mg/mL DMACA. All matrix solutions were sonicated for 10 min.

For derivatization, PBH was dissolved in chloroform to give a concentration of 25 μmol/mL. For experiments where mixtures of DHB/PBH were used, the matrix mixture was prepared in chloroform/methanol (1:1, *v/v*) giving a final concentration of 25 μmol/mL PBH and 0.5 mol/L DHB, respectively. Derivatization can be achieved by simple mixing of the derivatization reagent PBH, with the oxidized lipid directly in solution prior to spotting the mixture onto the MALDI target. The reaction occurs under ambient conditions and is very fast because an excess of PBH is used.

For MALDI-TOF MS, lipid extracts were mixed with the respective matrix solution. DHB and PBH were mixed with the samples 1:1, whereas samples were mixed with 9-AA in a ratio of 1:30, respectively (*v/v*). A volume of 0.75 μL was applied onto the MALDI target in duplicates, or 1 μL was applied in triplicates, if PBH was used. Samples were mixed 1:40 with DMACA and 0.5 μL of this mixture were applied to the MALDI target. The small amounts of samples mixed with either 9-AA or DMACA, respectively, were possible due to the considerable sensitivity of these matrices.

MALDI spectra were recorded on an Autoflex Speed mass spectrometer (Nd:YAG 355 nm “Smartbeam” laser, Bruker Daltonics GmbH, Bremen, Germany) in the positive and in the negative ion mode with a mass range of *m*/*z* 350–1200. Red phosphorus was used to calibrate the spectra.^34^

## Results and Discussion

To assess how different MALDI matrices influence the detection of certain oxPL in complex biological samples, we systematically compared the performance of different MALDI matrices for the analysis of selected lipid standards in rat liver extracts. The lipid standards were added to and then extracted from rat liver homogenates, which served as a representative biological specimen to mimic realistic tissue sample conditions. In the present study, commercially available LPC 17:0 and PC 16:0/9:0<oxo> were chosen as artificial oxPL while SM 18:1;O2/16:1 served as a nonoxidized artificial lipid with a choline headgroup for comparative purposes. These lipid standards were added to rat liver homogenates as these lipids were not detectable in the respective freshly prepared samples (Supporting Figure S1). Because the ionization efficiency of lipids in the MALDI process relies on the type of matrix used,^35^ lipid extracts were mixed separately with different matrix solutions as indicated above. For 9-AA and DMACA mixtures of 1:1 (matrix with the organic extract), analogous to DHB, resulted in very poor spectra (data not shown). However, dilutions of 1:30 and 1:40 sample to matrix, respectively, resulted in high quality spectra. Thus, these ratios between the lipid extract and the matrix solution were chosen for further experiments.

A concentration of 1 μg/mL of LPC 17:0 in 100 μL of liver homogenate, corresponding to a total amount of 3.75 ng on the MALDI target, was detectable by MALDI MS with DHB as the matrix (Figure 1A). Clear signals (three times signal-to-noise (*S*/*N*) ratio) for PC 16:0/9:0<oxo> were only visible at concentrations of at least 5 μg/mL (total amount: 18.75 ng, Figure 1B), leading to the conclusion that the detection of LPC on the respective MALDI mass spectrometer is five times more sensitive compared to the aldehyde using DHB. The spectra showed no indication of artificial LPC formation from the added PC 16:0/9:0<oxo> standard or nonoxidized endogenous PC molecules (see Supporting Figure S2), which is a known side effect.^36^ One potential reason is that the laser fluence was kept as low as possible to avoid unnecessary fragmentation. However, using DHB, the *S*/*N* ratio of endogenous LPC molecules fluctuated between the measurements. Interestingly, the extent of proton and sodium adduct formation differed between the PC-containing lipid classes. While signal intensities for proton adducts of LPC and PC 16:0/9:0<oxo> were much higher compared to the sodium signals, the difference was less pronounced for intact PC molecules. This is an indication that Na^+^ is primarily bound to the apolar site of the lipid.

**Figure 1:**
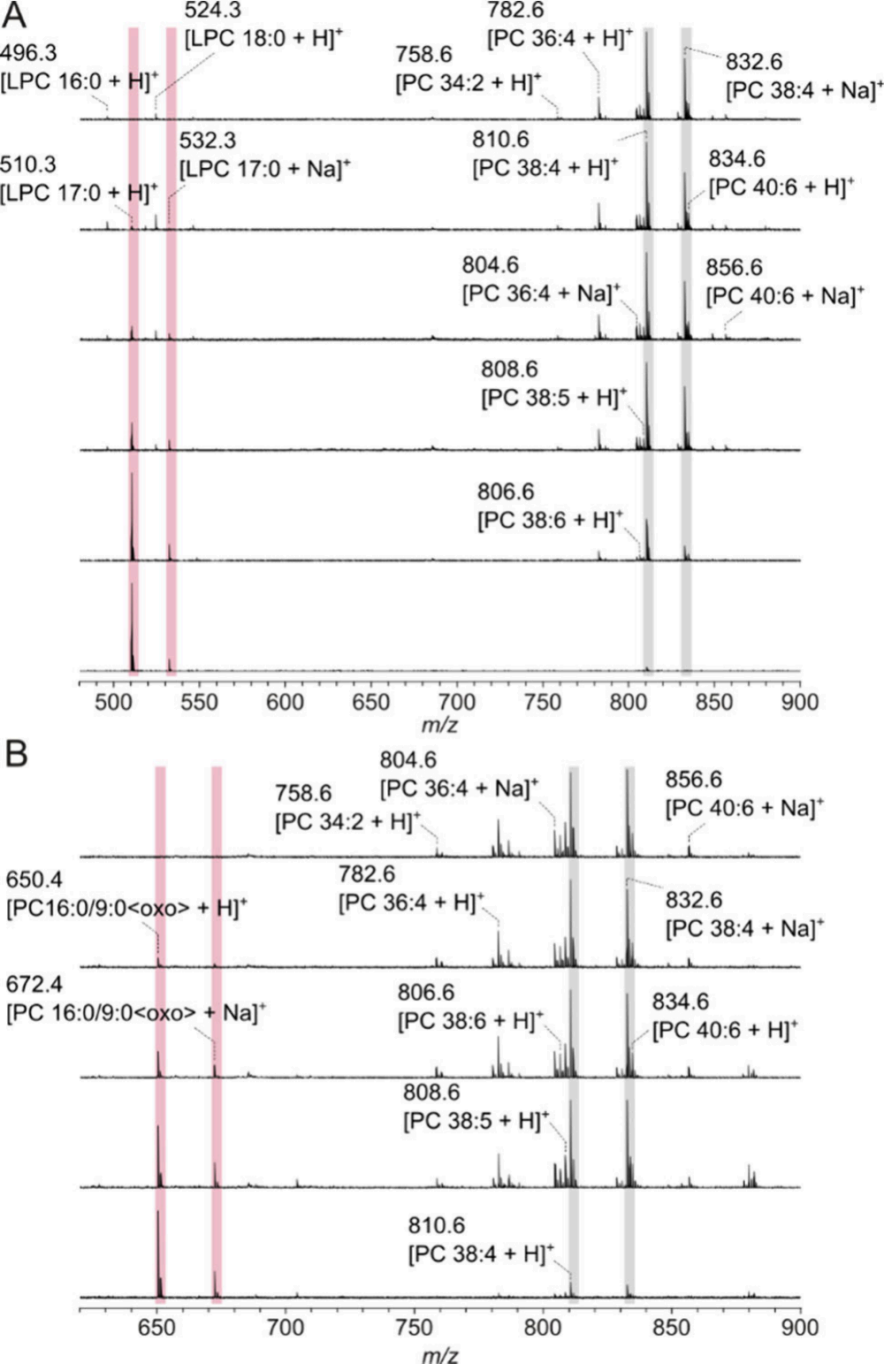
Positive ion MALDI-TOF mass spectra of rat liver extracts acquired with 2,5-dihydroxybenzoic acid (DHB, 0.5 M in methanol) as the matrix. The lipid extract was mixed with DHB in a ratio of 1:1 (*v/v*) and 0.75 μL of the mixture were applied onto the MALDI target. Solutions of LPC 17:0 (A) and PC 16:0/9:0<oxo> (B) were mixed with rat liver extracts to obtain the different concentrations highlighted in pale red. The signals of PC 38:4, the most abundant phospholipid in rat liver, are highlighted in gray.

In contrast to DHB, 9-AA allowed for a far more sensitive detection of LPC 17:0 and PC 16:0/9:0<oxo>. Lipid extracts were mixed with 9-AA in a ratio of 1:30 (*v/v*) and lipids were detectable at concentrations of 1 μg/mL for LPC and 5 μg/mL for the aldehyde (Figure 2), corresponding to a total amount of 0.25 ng and 1.25 ng of lipid, respectively. This is a 15-fold increased sensitivity of 9-AA compared to DHB. As with DHB, LPC was five times more sensitively detected than PC 16:0/9:0<oxo> using 9-AA. Another advantage of the use of 9-AA is the nearly exclusive presence of H^+^ adducts while Na^+^ adducts are absent because 9-AA has a high affinity to alkali ions.^32^ Thus, there is no signal splitting due to the formation of alkali adducts and, therefore, a higher detection sensitivity compared to matrices that generate proton as well as alkali adducts.^37^ Furthermore, the *S*/*N* ratio of endogenous LPC ions was higher and its determination more accurate with 9-AA compared to DHB (DHB, *S*/*N* LPC 16:0:2.7–9.5; LPC 18:0:2.8–10.8, 9-AA; *S*/*N* LPC 16:0:6–9.3, LPC 18:0:9.8–17.5, Supporting Figure S2). This effect is even more remarkable, given that the samples were much more diluted with 9-AA than with DHB. Again, there was no indication of additional LPC formation from the artificially added aldehyde and endogenous PC molecules.

**Figure 2:**
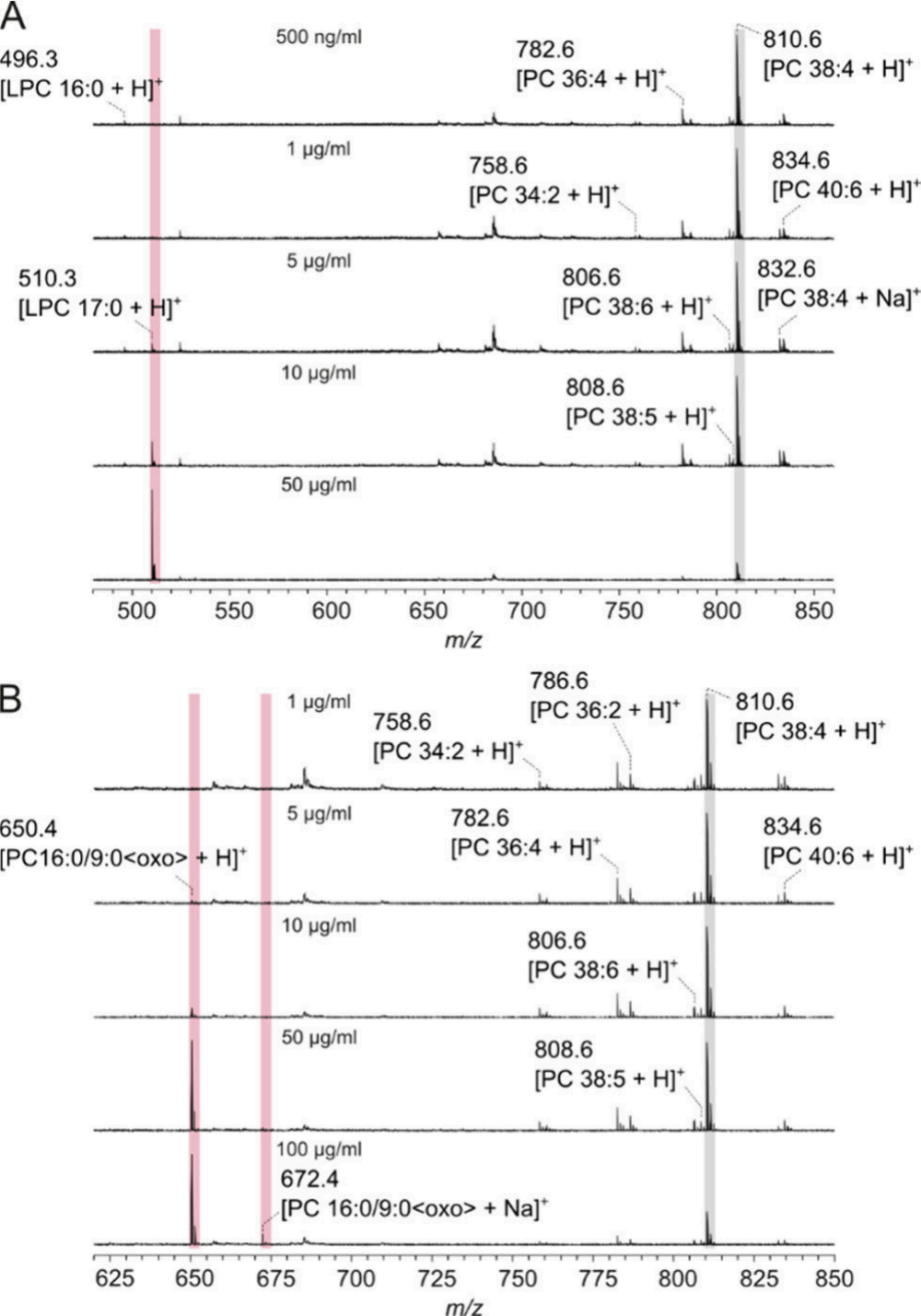
Positive ion MALDI mass spectra of rat liver extracts with 9-aminoacridine (9-AA) as the matrix. The lipid extract was mixed with 9-AA (10 mg/mL in acetonitrile/2-propanol, 4:6, *v/v*) in a ratio of 1:30 (*v/v*) and 0.75 μL of the mixture were applied onto the MALDI target. LPC 17:0 (A) and PC 16:0/9:0<oxo> (B) were mixed with rat liver extracts to get different concentrations of the artificial lipids highlighted in pale red. The signals of PC 38:4, the most abundant phospholipid in rat liver, are highlighted in gray. The broad peak at *m*/*z* 685 could not be assigned.

The photophysical properties of DMACA in different solvents have already been tested^38^ and revealed that the absorption maxima are close to the excitation wavelength of the Nd:YAG laser of 355 nm. DMACA was shown to be a suitable matrix substance for MALDI MSI only recently.^33^ A concentration of 5 mg/mL DMACA dissolved in acetonitrile/THF (1:3, *v/v*) gave the best results for investigations in the positive (Supporting Figure 3) and the negative ion mode (Supporting Figure 4) and was used for further experiments. The sensitivity of DMACA for LPC was even higher, as lipid extracts had to be mixed at a 1:40 ratio (*v/v*) with DMACA to generate mass spectra with sufficient *S*/*N* ratios (Figure 3). While LPC was already detected in the 1 μg/mL dilution (0.125 ng), PC 16:0/9:0<oxo> was only detectable at 50 μg/mL (6.25 ng) and, thus, 50-fold less sensitively detectable than LPC. This could account for a serious discrimination of oxPL, such as aldehydes, by DMACA. However, the *S*/*N* ratio of endogenous LPC ions was stable between the measurements, again demonstrating that there was no additional LPC formation resulting from the MALDI ionization process and/or in source decay (*S*/*N* LPC 16:0:2–4, LPC 18:0:6.3–8.8, Supporting Figure S2). Astonishingly, DMACA resulted in different yields of proton and sodium adducts of the different PC species. Higher proportions of proton adducts were detected for LPC, whereas proton and sodium adducts were approximately balanced for PC 16:0/9:0<oxo> and sodium adducts were even preferentially generated for intact PC molecules, such as PC 38:4. Again, this is an indication that alkali metal ions are preferentially bound to the apolar residues of the analyte.

**Figure 3:**
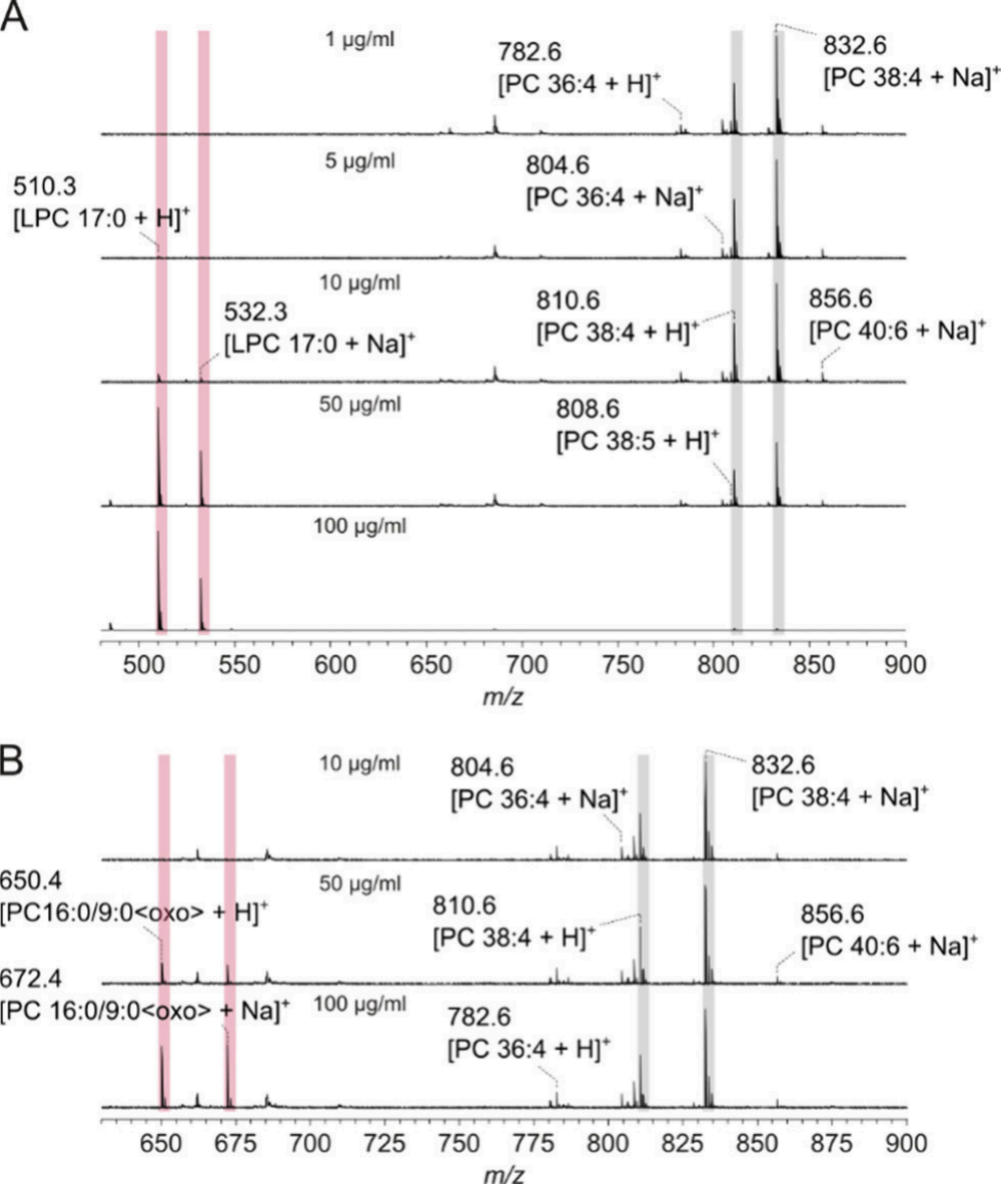
Positive ion MALDI-TOF mass spectra of rat liver extracts with 4-(dimethylamino)-cinnamic acid (DMACA) as the matrix. The lipid extract was mixed with DMACA (5 mg/mL in acetonitrile/THF, 1:3, *v/v*) in a ratio of 1:40 (*v/v*), and 0.5 μL of the mixture was applied onto the MALDI target. LPC 17:0 (A) and PC 16:0/9:0<oxo> (B) were mixed with rat liver extracts to get different concentrations of the artificial lipids highlighted in pale red. The signals of PC 38:4, the most abundant phospholipid in rat liver, are highlighted in gray.

In order to compare the performance of the matrices toward other (nonoxidized) lipids with a choline headgroup, SM 18:1;O2/16:1 was added to the liver extracts. Using DHB, this sphingolipid was detected at a minimum concentration of approximately 5 μg/mL (total amount: 18.75 ng). Just as for LPC and PC 16:0/9:0<oxo>, 9-AA and DMACA resulted in a more sensitive detection of SM 18:1;O2/16:1 compared to DHB (Supporting Figure 5).

To depict the efficiency of the different matrices for the added nonendogenous lipids, the ratios of the integrated peak areas of the artificial lipid standards to the most abundant endogenous PC in rat liver tissue, PC 38:4, were calculated and plotted against the total amount of the added lipid standards onto the MALDI target (Figure 4). A logarithmic scale was chosen to ensure reasonable visibility of all data points. For all three artificially added lipids, DHB was the least sensitive matrix. In contrast, amounts of less than 1 ng of lipid were already detectable with 9-AA and/or DMACA.

**Figure 4:**
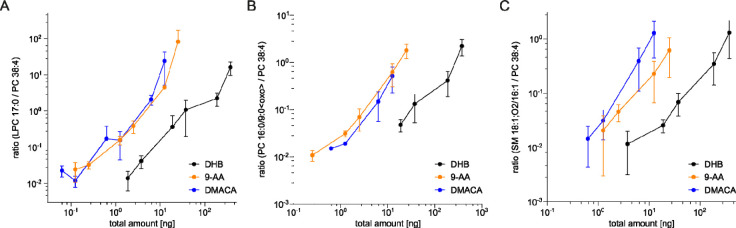
Ratios of the integrated peak areas of artificially added lipids to endogenous PC 38:4 plotted against the total amounts of artificially added lipids on the MALDI target. Mass spectra were recorded in the positive ion mode using DHB, 9-AA, or DMACA as the matrix (cf. main text). PC 38:4 is the most abundant phosphatidylcholine in rat liver extracts and was used as reference. Ratios depicted represent (A) LPC 17:0/PC 38:4, (B) PC 16:0/9:0<oxo>/PC 38:4, (C) SM 18:1:O2/16:1/PC 38:4. For the calculations, the integrated peak areas of the H^+^ and Na^+^ adducts were summed. Dots present mean values of *n* = 4 measurements, and error bars present mean ± standard deviation. Data are depicted in log scale.

As depicted in Figure 4, DMACA and 9-AA allow for a highly sensitive detection of LPC. However, detection of PC 16:0/9:0<oxo> was at least five times less sensitive in comparison to LPC for all of the three investigated matrices, DHB, 9-AA, and DMACA. Presumably, ion suppression might also affect the detectability of certain oxPL, such as truncated aldehydes, in mixtures. Thus, due to the low abundance and the transient nature of truncated oxidized lipids and their inferior detectability in comparison to “native” lipids, improved MS methods for their analysis are urgently required.

One potential way to improve the sensitivity and selectivity for the detection of oxidized lipids is the use of suitable chemical derivatization reactions. Different reactive matrices possessing ionization-enhancing aromatic groups, have already been applied in MALDI MS for on-target and on-tissue derivatization.^27,28,24^ A well-known textbook reaction is the derivatization of aldehydes with 2,4-dinitrophenylhydrazine (DPNH). Similarly, PBH, a common fluorescence labeling reagent, can be used for detecting carbonyl compounds, such as lipid peroxidation-derived PL aldehydes.^39^ PBH possesses a significant UV absorption and, thus, enables the application of low MALDI laser fluences while increasing the ion yields of derivatized compounds. On-target derivatization with PBH can be easily achieved by the simple mixing of the reactive matrix with the sample and leads to a characteristic mass shift of +284 Da through the addition of the matrix and the elimination of water (Figure 5A and Supporting Figure 6). In our experiments, we used an excess of PBH, and thus, the aldehyde was always completely converted. We did not evaluate the derivatization of ketones. However, it is assumed that the reaction would be much slower because ketones are less reactive than aldehydes. The product of the derivatization reaction of PC 16:0/9:0<oxo> and PBH was systematically named PC 16:0/9:0<PBH> and can be detected in concentrations as low as 0.5 μg/mL. In comparison to DHB alone, where PC 16:0/9:0<oxo> can only be detected in concentrations higher than 5 μg/mL, derivatization with PBH results in a ten times lower detection limit, thereby reaching potentially relevant biological concentrations.^40^ The peak ratio between PC 16:0/9:0<PBH> and the endogenous PC with the highest concentration in rat liver extracts, PC 38:4, in PBH/DHB is substantially higher than the ratio of PC 16:0/9:0<oxo> to PC 38:4 in DHB (Figure 5B). This demonstrates that PBH derivatization increases the ionization efficiency of oxPC and probably other oxPL with reactive carbonyl groups. The ratio of derivatized and native PC 16:0/9:0<oxo> to PC 38:4 at 100 μg/mL was not significantly different. This can arguably be attributed to the high concentration of supplemented PC 16:0/9:0<oxo>, by which potential ion suppression effects occur and detector saturation is reached. Additionally, the same sample that had already been prepared on the target was remeasured the following day, with no noticeable signal decrease. Spectra recorded after different exposure times of the target to high vacuum within the mass spectrometer were virtually identical indicating good vacuum stability of the reactive matrix PBH.

**Figure 5:**
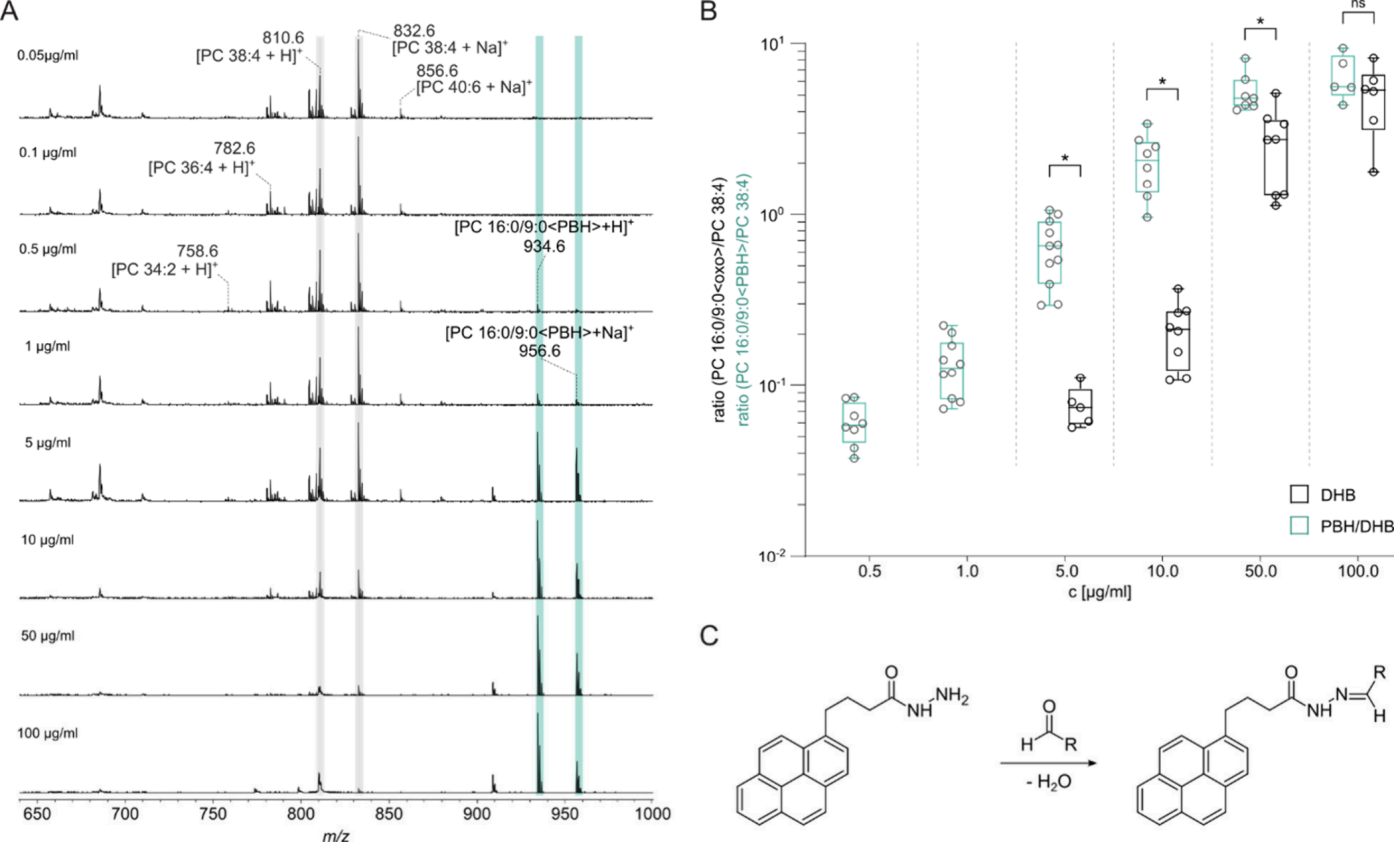
On-target derivatization with 1-pyrenebutyric hydrazide (PBH) enhances the detectability of lipid peroxidation-derived phospholipid aldehydes. PBH/DHB (1:1, *v/v*) was used as the matrix. Concentrations refer to the artificially added oxPL standard PC 16:0/9:0<oxo>. (A) Positive ion MALDI-TOF mass spectra. (B) Box plot showing differences in ionization efficiencies using DHB or PBH/DHB (1:1, *v/v*), respectively. The *p* values were calculated by *t* test (asterisk * indicates *p* < 0.05; ns, not significant). Boxplot elements represent the following: centerline, mean; box limits, 25% and 75% quartiles; vertical lines connect minimum and maximum values. Dots represent independent measurements. (C) Chemical reaction of PBH with an aldehyde, such as PC 16:0/9:0<oxo>.

Our initial experiments were conducted with commercially available oxidized lipid standards and are therefore limited to PC as so far the only commercially available oxidized compounds. The herein proposed derivatization approach for the improved detection of oxPL, however, can be extended to other lipid classes, as the hydrazine group of PBH reacts with the generated aldehyde group under water loss (Figure 5C and Supporting Figure 6B). In order to test the applicability of this approach to other lipid classes, oxidized PE 18:0/20:4, for which no standards are commercially available, was generated in-house by mild *in vitro* oxidation using copper/ascorbate. In many mammalian tissues, PE is the second highest abundant PL class, among which PE 18:0/20:4 is typically one of the PL species with the highest concentration.^41,42^ Since PE is predominantly located on the inner layer of cell membranes,^43^ they are also frequently subjected to lipid peroxidation, and thus, the arachidonic acid esterified within PE 18:0/20:4 can be easily oxidized *in vivo*. During the in-house *in vitro* copper/ascorbate oxidation of PE 18:0:20:4, hydroxyl and partially hydroperoxyl radicals are generated, mimicking naturally occurring oxidation conditions.^30^ The main peaks in the corresponding spectra of *in vitro* oxidized PE 18:0/20:4 stem from the remaining nonoxidized PE standard (Figure 6 and Table 1) indicating that the yield of oxidized products is rather poor. Some peaks can be attributed to characteristic oxidation products, such as the corresponding lyso species LPE 18:0/0:0 or LPE 0:0/20:4, hydroxylated species PE 18:0/20:4<OH> or the carboxylic acid PE 18:0/5:0<COOH>. However, when using only the conventional MALDI matrices DHB or 9-AA, no signal corresponding to the expected aldehyde species PE 18:0/5:0<oxo> can be detected (Figure 6A,B, upper panels). Remarkably, however, the aldehyde can be easily detected subsequent to PBH derivatization. When PBH is used as the reactive matrix, a signal with *m*/*z* = 908.5 in the positive ion mode (Figure 6A, lower panel) and *m*/*z* = 884.5 in the negative ion mode (Figure 6B, lower panel) can be detected. This corresponds to PE 18:0/5:0<PBH> subsequent to derivatization with PBH and is obvious from the characteristic mass shift of +284 Da. Derivatization with PBH increases the detection sensitivity of this aldehyde in the negative ion mode to such an extent that the signal intensities of the educt PE 18:0/20:4 and the derivatized oxidation product PE 18:0/5:0<PBH> possess comparable intensities. In comparison to the spectra recorded in the presence of 9-AA, the signal intensities of LPE and the carboxylic acid PE species are also increased, which arguably makes PBH a promising matrix for the detection of lipid oxidation products with reactive carbonyl groups. Another advantage is the nearly complete absence of proton adducts if PBH is used, making peak assignments (particularly in complex mixtures and on mass spectrometers with poor resolution) more straightforward.

**Figure 6:**
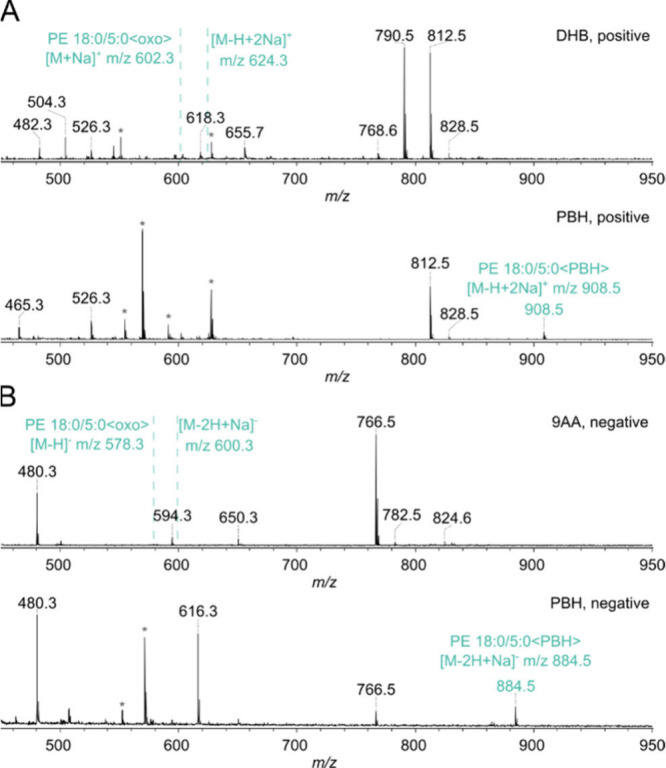
*In vitro* oxidized PE 18:0/20:4 monitored by MALDI-TOF MS in the positive (A) and the negative ion mode (B). The PE standard was oxidized *in vitro* by copper/ascorbate. The aldehyde obtained by oxidation cannot be detected by the use of conventional MALDI matrices, such as DHB or 9-AA. However, the aldehyde can be easily detected subsequent to PBH derivatization. Peaks originating from the matrix are marked with an asterisk.

**Table 1:** Mass List of *In Vitro* Oxidized PE 18:0/20:4 Detected by MALDI-TOF MS^a^

	[M – H]^−^	[M – 2H + Na]^−^	[M + H]^+^	[M + Na]^+^	[M – H + 2Na]^+^
PE 18:0/20:4	766.5	788.5^nd^	768.6	790.5	812.5
PE 18:0/20:4<OH>	782.5	804.5^nd^	784.5^nd^	806.5	828.5
PE 18:0/5:0<oxo>	578.3^nd^	600.3^nd^	580.4^nd^	602.3^nd^	624.3^nd^
PE 18:0/5:0<COOH>	594.3	616.3	596.4^nd^	618.3	640.3
LPE 18:0/0:0	480.3	502.3^nd^	482.3	504.3	526.3
LPE 0:0/20:4	500.3	522.3^nd^	502.3^nd^	524.3^nd^	546.3^nd^
PE 18:0/5:0<PBH>	862.5^nd^	884.5	864.5^nd^	886.5^nd^	908.5

a Not all ions can be detected with all three matrices discussed herein. Ions that were not detectable or had an *S*/*N* ratio of less than 3 are labeled with nd (not detectable).

PBH can be used in combination with DHB to increase the ion yield of truncated aldehyde lipids for detection in positive ion mode. The use of PBH alone in the positive ion mode did not show significant advantages and resulted in poor spectra compared to the combination of DHB and PBH (Supporting Figure 6A). PBH can also be combined with 9-AA for the detection of lipids in positive or negative ion mode. A combination of PBH and 9-AA did not show any advantage over DHB/PBH in positive ion mode. Using PBH alone for the detection in the negative ion mode resulted in high quality spectra and omits the need for a combination with another matrix for lipid analysis as well. Additionally, PBH is particularly useful in the negative ion mode, where SM and PC may be also detected (subsequent to the loss of a methyl group) in addition to more acidic lipids such as PI and PS (Supporting Figure 6). The combination of DMACA and PBH was also attempted but unsuccessful due to the incompatibility of the solubility of the respective compounds in the tailored organic solvent mixtures.

## Conclusions

Our findings emphasize the critical role of careful matrix selection in the interpretation of MALDI data. The absence of detectable signals does not necessarily indicate the absence of a compound; rather, different matrices influence the ionization efficiency of various compounds, even those with similar structures, as demonstrated with three PC headgroup-containing PL in this study. Among the tested matrices, 9-AA and DMACA enabled the more sensitive detection of PC-containing lipids compared to DHB. All three matrices, DHB, 9-AA, and DMACA, were more sensitive toward detecting LPC in comparison to truncated aldehyde species presumably due to the smaller molecular weight of LPC.

Derivatization with PBH significantly enhanced the sensitivity for the detection of truncated aldehyde-containing oxPL. This makes PBH particularly valuable for identifying low-abundance, potentially suppressed oxPL species. Since these oxPL result from nonenzymatic oxidation, PBH-based detection can be especially beneficial in the analysis of tissues affected by oxidative stress, i.e., in inflammatory diseases. Therefore, PBH shows considerable promise for future applications in MALDI MSI, considering that matrix deposition results in a sufficient derivatization reaction, which has yet to be evaluated.

## Supplementary Material



## Notes

Supporting Figures S1–S6 showing MALDI-TOF mass spectra of freshly prepared organic extracts from rat liver analyzed with the three different matrix substances; signal-to-noise ratios of the proton adducts of two LPC proton adducts and PC 16:0/9:0<oxo> under increasing PC 16:0/9:0<oxo> amounts; performance of DMACA as MALDI matrix for liver samples applied at different concentrations dissolved in different solvents; MALDI-TOF mass spectra of rat liver extracts with different amounts of artificially added SM 18:1;O2/16:1; MALDI TOF mass spectra of derivatized PC 16:0/5:0<PBH> (PDF)

## Abbreviations

9-AA: 9-aminoacridine
ABC: ammonium bicarbonate
DHB: 2,5-dihydroxybenozoic acid
DMACA: 4-(dimethylamino)­cinnamic acid
Lp­(a): lipoprotein a
LPC: lysophosphatidylcholine
LPE: lysophosphatidylethanolamine
MALDI-TOF MS: matrix-assisted laser desorption/ionization time-of-flight mass spectrometry
MSI: mass spectrometry imaging
oxPC: oxidized phosphatidylcholine
oxPL: oxidized phospholipid
PBH: 1-pyrenebutyric hydrazide
PC: phosphatidylcholine
PE: phosphatidylethanolamine
SM: sphingomyelin
THF: tetrahydrofuran

## References

[1] Eibl H. (1984). Phospholipids
as Functional Constituents of Biomembranes. Angew. Chem., Int. Ed. Engl..

[2] Turk J., Wolf B. A., Lefkowith J. B., Stump W. T., McDaniel M. L. (1986). Glucose-Induced
Phospholipid Hydrolysis in Isolated Pancreatic Islets: Quantitative
Effects on the Phospholipid Content of Arachidonate and other Fatty
Acids. Biochim. Biophys. Acta.

[3] Wiesner P., Leidl K., Boettcher A., Schmitz G., Liebisch G. (2009). Lipid Profiling
of FPLC-Separated Lipoprotein Fractions by Electrospray Ionization
Tandem Mass Spectrometry. J. Lipid Res..

[4] Gardner H. W. (1989). Oxygen
Radical Chemistry of Polyunsaturated Fatty Acids. Free Radic. Biol. Med..

[5] Fuchs B., Bresler K., Schiller J. (2011). Oxidative Changes of
Lipids Monitored
by MALDI MS. Chem. Phys. Lipids.

[6] Else P. L., Kraffe E. (2015). Docosahexaenoic and
Arachidonic Acid Peroxidation:
It’’s a Within Molecule Cascade. Biochim. Biophys. Acta.

[7] Engel K. M., Schiller J. (2017). A Comparison of PC Oxidation Products
as Detected by
MALDI-TOF and ESI-IT Mass Spectrometry. Chem.
Phys. Lipids.

[8] Haller E., Stübiger G., Lafitte D., Lindner W., Lämmerhofer M. (2014). Chemical Recognition
of Oxidation-Specific Epitopes in Low-Density Lipoproteins by a Nanoparticle-based
Concept for Trapping, Enrichment, and Liquid Chromatography-Tandem
Mass Spectrometry Analysis of Oxidative Stress Biomarkers. Anal. Chem..

[9] Ogita T., Tanaka Y., Nakaoka T., Matsuoka R., Kira Y., Nakamura M., Shimizu T., Fujita T. (1997). Lysophosphatidylcholine
Transduces Ca^2+^ Signaling via the Platelet-Activating Factor
Receptor in Macrophages. Am. J. Physiol..

[10] Tsimikas S., Viney N. J., Hughes S. G., Singleton W., Graham M. J., Baker B. F., Burkey J. L., Yang Q., Marcovina S. M., Geary R. S., Crooke R. M., Witztum J. L. (2015). Antisense
Therapy Targeting Apolipoprotein­(a): a Randomised, Double-Blind, Placebo-Controlled
Phase 1 Study. Lancet.

[11] Capoulade R., Chan K. L., Yeang C., Mathieu P., Bossé Y., Dumesnil J. G., Tam J. W., Teo K. K., Mahmut A., Yang X., Witztum J. L., Arsenault B. J., Després J.-P., Pibarot P., Tsimikas S. (2015). Oxidized Phospholipids,
Lipoprotein­(a), and Progression of Calcific Aortic Valve Stenosis. J. Am. Coll. Cardiol..

[12] Stiuso P., Scognamiglio I., Murolo M., Ferranti P., De Simone C., Rizzo M. R., Tuccillo C., Caraglia M., Loguercio C., Federico A. (2014). Serum Oxidative Stress Markers and
Lipidomic Profile
to Detect NASH Patients Responsive to an Antioxidant Treatment: a
Pilot Study. Oxid. Med. Cell Longevity.

[13] Sun X., Seidman J. S., Zhao P., Troutman T. D., Spann N. J., Que X., Zhou F., Liao Z., Pasillas M., Yang X., Magida J. A., Kisseleva T., Brenner D. A., Downes M., Evans R. M., Saltiel A. R., Tsimikas S., Glass C. K., Witztum J. L. (2020). Neutralization
of Oxidized Phospholipids Ameliorates
Non-alcoholic Steatohepatitis. Cell Metab..

[14] Fujii H., Ikura Y., Arimoto J., Sugioka K., Iezzoni J. C., Park S. H., Naruko T., Itabe H., Kawada N., Caldwell S. H., Ueda M. (2009). Expression
of Perilipin and Adipophilin
in Nonalcoholic Fatty Liver Disease; Relevance to Oxidative Injury
and Hepatocyte Ballooning. J. Atheroscler. Thromb..

[15] Younossi Z. M., Golabi P., Paik J. M., Henry A., van Dongen C., Henry L. (2023). The Global Epidemiology
of Nonalcoholic Fatty Liver Disease (NAFLD)
and Nonalcoholic Steatohepatitis (NASH): A Systematic Review. Hepatology.

[16] Kagan V. E., Chu C. T., Tyurina Y. Y., Cheikhi A., Bayir H. (2014). Cardiolipin
Asymmetry, Oxidation and Signaling. Chem. Phys.
Lipids.

[17] Lands W. E. (1960). Metabolism
of Glycerolipids. 2. The Enzymatic Acylation of Lysolecithin. J. Biol. Chem..

[18] O’Donnell V. B. (2022). New Appreciation
for an Old Pathway: the Lands Cycle Moves into New Arenas in Health
and Disease. Biochem. Soc. Trans..

[19] Prabutzki P., Schiller J., Engel K. M. (2024). Phospholipid-Derived
Lysophospholipids
in (Patho)­physiology. Atherosclerosis.

[20] Wölk M., Prabutzki P., Fedorova M. (2023). Analytical Toolbox to Unlock the
Diversity of Oxidized Lipids. Acc. Chem. Res..

[21] Damiani T., Bonciarelli S., Thallinger G. G., Koehler N., Krettler C. A., Salihoǧlu A. K., Korf A., Pauling J. K., Pluskal T., Ni Z., Goracci L. (2023). Software and Computational Tools for LC-MS-Based Epilipidomics:
Challenges and Solutions. Anal. Chem..

[22] Leopold J., Popkova Y., Engel K. M., Schiller J. (2018). Visualizing Phosphatidylcholine
via Mass Spectrometry Imaging: Relevance to Human Health. Expert Rev. Proteomics.

[23] Leopold J., Popkova Y., Engel K. M., Schiller J. (2018). Recent Developments
of Useful MALDI Matrices for the Mass Spectrometric Characterization
of Lipids. Biomolecules.

[24] Stübiger G., Wuczkowski M., Bicker W., Belgacem O. (2014). Nanoparticle-Based
Detection of Oxidized Phospholipids by MALDI Mass Spectrometry: Nano-MALDI
Approach. Anal. Chem..

[25] Bacellar I. O. L., Oliveira M. C., Dantas L. S., Costa E. B., Junqueira H. C., Martins W. K., Durantini A. M., Cosa G., Di Mascio P., Wainwright M., Miotto R., Cordeiro R. M., Miyamoto S., Baptista M. S. (2018). Photosensitized
Membrane Permeabilization Requires
Contact-Dependent Reactions between Photosensitizer and Lipids. J. Am. Chem. Soc..

[26] Zhang Y., Iwamoto T., Radke G., Kariya Y., Suzuki K., Conrad A. H., Tomich J. M., Conrad G. W. (2008). On-Target Derivatization
of Keratan Sulfate Oligosaccharides with Pyrenebutyric Acid Hydrazide
for MALDI-TOF/TOF-MS. J. Mass Spectrom..

[27] Borisov R. S., Matveeva M. D., Zaikin V. G. (2023). Reactive
Matrices for Analytical
Matrix-Assisted Laser Desorption/Ionization (MALDI) Mass Spectrometry. Crit. Rev. Anal. Chem..

[28] Harkin C., Smith K. W., Cruickshank F. L., Logan Mackay C., Flinders B., Heeren R. M. A., Moore T., Brockbank S., Cobice D. F. (2022). On-Tissue Chemical Derivatization
in Mass Spectrometry
Imaging. Mass Spectrom. Rev..

[29] Flinders B., Morrell J., Marshall P. S., Ranshaw L. E., Clench M. R. (2015). The Use
of Hydrazine-Based Derivatization Reagents for Improved Sensitivity
and Detection of Carbonyl Containing Compounds Using MALDI-MSI. Anal. Bioanal. Chem..

[30] Lange M., Wagner P. V., Fedorova M. (2021). Lipid Composition Dictates
the Rate
of Lipid Peroxidation in Artificial Lipid Droplets. Free Radical Res..

[31] Schiller J., Süss R., Fuchs B., Müller M., Petković M., Zschörnig O., Waschipky H. (2007). The Suitability
of Different DHB Isomers as Matrices for the MALDI-TOF MS Analysis
of Phospholipids: Which Isomer for What Purpose?. Eur. Biophys. J..

[32] Sun G., Yang K., Zhao Z., Guan S., Han X., Gross R. W. (2008). Matrix-Assisted Laser Desorption/Ionization Time-of-Flight
Mass Spectrometric Analysis of Cellular Glycerophospholipids Enabled
by Multiplexed Solvent Dependent Analyte-Matrix Interactions. Anal. Chem..

[33] Dufresne M., Migas L., Djambazova K., Colley M., van de Plas R., Spraggins J. (2025). Aminated Cinnamic Acid Analogs as Dual Polarity Matrices
for High Spatial Resolution MALDI Imaging Mass Spectrometry. Anal. Chim. Acta.

[34] Sládková K., Houska J., Havel J. (2009). Laser Desorption Ionization of Red
Phosphorus Clusters and their Use for Mass Calibration in Time-of-Flight
Mass Spectrometry. Rapid Commun. Mass Spectrom..

[35] Estrada R., Yappert M. C. (2004). Alternative Approaches
for the Detection of Various
Phospholipid Classes by Matrix-Assisted Laser Desorption/Ionization
Time-of-Flight Mass Spectrometry. J. Mass Spectrom..

[36] Stübiger G., Belgacem O., Rehulka P., Bicker W., Binder B. R., Bochkov V. (2010). Analysis of Oxidized
Phospholipids by MALDI Mass Spectrometry
Using 6-Aza-2-Thiothymine Together with Matrix Additives and Disposable
Target Surfaces. Anal. Chem..

[37] Zschörnig O., Richter V., Rassoul F., Süß R., Arnold K., Schiller J. (2006). Analysis of Human Blood
Plasma by
MALDI-TOF MS—Evaluation of Critical Parameters. Anal. Lett..

[38] Bangal P. R., Chakravorti S. (1998). Photophysics of 4-Dimethylamino Cinnamic
Acid in Different
Environments. J. Photochem. Photobiol., A.

[39] Bacellar I. O. L., Baptista M. S. (2019). Mechanisms of Photosensitized
Lipid Oxidation and Membrane
Permeabilization. ACS Omega.

[40] Oskolkova O. V., Afonyushkin T., Preinerstorfer B., Bicker W., von Schlieffen E., Hainzl E., Demyanets S., Schabbauer G., Lindner W., Tselepis A. D., Wojta J., Binder B. R., Bochkov V. N. (2010). Oxidized Phospholipids are More Potent Antagonists
of Lipopolysaccharide than Inducers of Inflammation. J. Immunol..

[41] Lange M., Angelidou G., Ni Z., Criscuolo A., Schiller J., Blüher M., Fedorova M. (2021). AdipoAtlas: A Reference
Lipidome for Human White Adipose Tissue. Cell
Rep. Med..

[42] Prabutzki P., Wölk M., Böttner J., Ni Z., Werner S., Thiele H., Schiller J., Büttner P., Schlotter F., Fedorova M. (2025). Sex-Specific Lipidomic Signatures
in Aortic Valve Disease Reflect Differential Fibro-Calcific Progression. Nat. Commun..

[43] Calderón R. O., DeVries G. H. (1997). Lipid Composition
and Phospholipid Asymmetry of Membranes
from a Schwann Cell Line. J. Neurosci. Res..

