# Supplementary material for: MALDI-TOF MS Detection of Oxidized Major Phospholipids in Biological Samples Using Conventional Matrices and 1‑Pyrenebutyric Hydrazide
Source: J Am Soc Mass Spectrom. 2025 Sep 16;36(10):2197–205. doi: 10.1021/jasms.5c00196 (PMC12492386; doi:10.1021/jasms.5c00196)
Supplement: Supplementary file 1 [file js5c00196_si_001.pdf]

# Supporting Information

## MALDI-TOF MS Detection of Oxidized Major Phospholipids in Biological Samples Using Conventional Matrices and 1-Pyrenebutyric Hydrazide

*Patricia Prabutzki, Jürgen Schiller and Kathrin M. Engel**

Institute of Medical Physics and Biophysics, Faculty of Medicine, Leipzig University,

Härtelstrasse 16-18, 04107 Leipzig, Germany

corresponding author: Kathrin M. Engel, kathrin.engel@medizin.uni-leipzig.de

Supporting Figures: MALDI-TOF mass spectra of freshly prepared organic extracts from rat liver analyzed with the three different matrix substances; Signal-to-noise ratios of the proton adducts of two LPC proton adducts and PC 16:0/9:0<oxo> under increasing PC 16:0/9:0<oxo> amounts; Performance of DMACA as MALDI matrix for liver samples applied at different concentrations dissolved in different solvents; MALDI-TOF mass spectra of rat liver extracts with different amounts of artificially added SM 18:1;O2/16:1; MALDI TOF mass spectra of derivatized PC 16:0/5:0<PBH> (DOC).

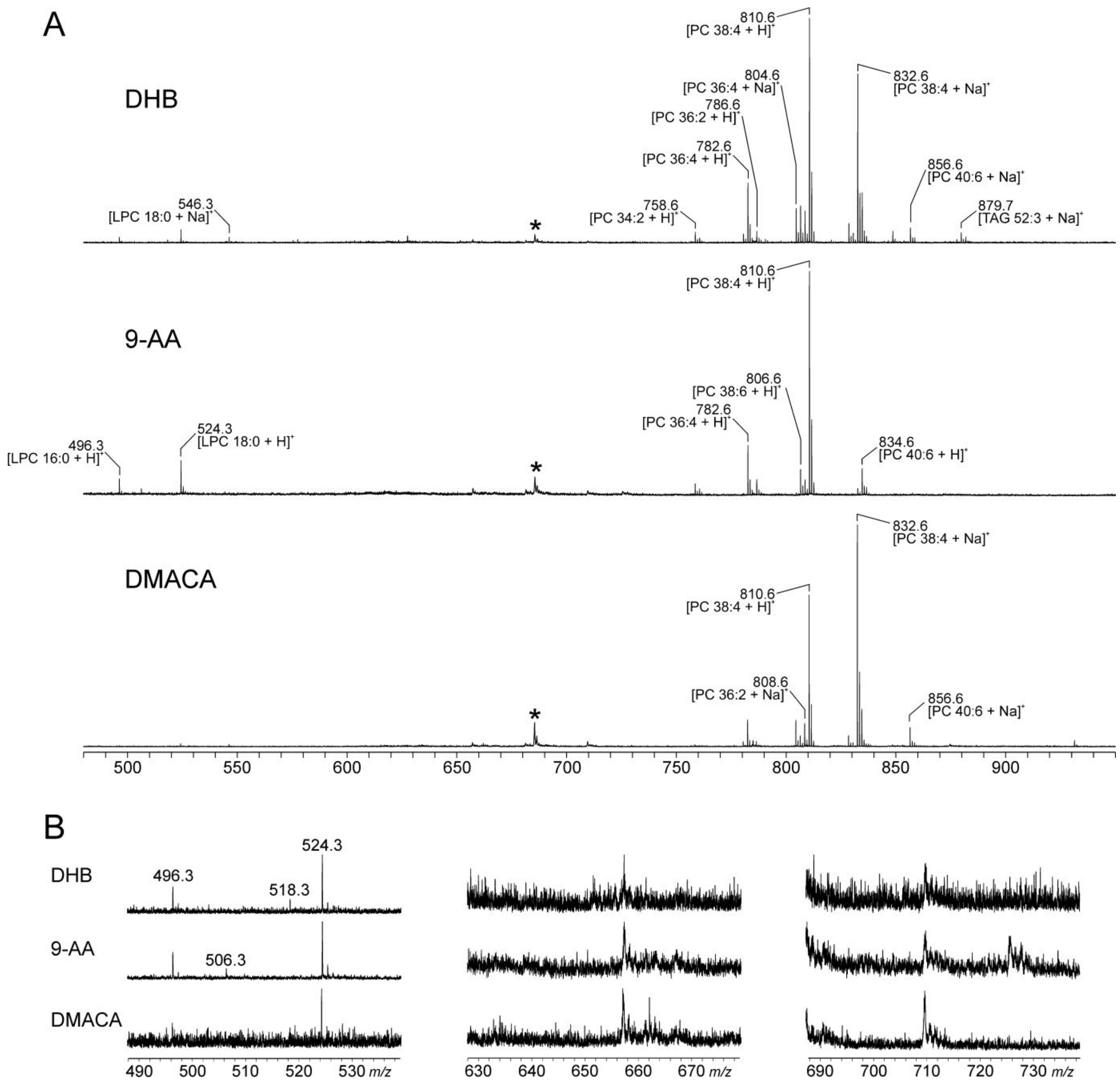


**Supporting Figure S1.** Positive ion MALDI-TOF mass spectra of freshly prepared organic extracts from rat liver. Organic extracts were mixed 1:1, 1:30 and 1:40 with DHB (0.5 M in methanol), 9-AA (10 mg/ml in acetonitrile/2-propanol, 4:6) and DMACA (5 mg/ml in acetonitrile/THF) matrix solutions, respectively. (**A**) MALDI mass spectra recorded from *m/z* 480 to 950, corresponding to the range of lysophosphatidylcholines (LPC) and phosphatidylcholines (PC). (**B**) Emphasized region of the respective *m/z* ranges of LPC (left), truncated PC (middle) and sphingomyelin species (right). The asterisk denotes a so far unknown peak.

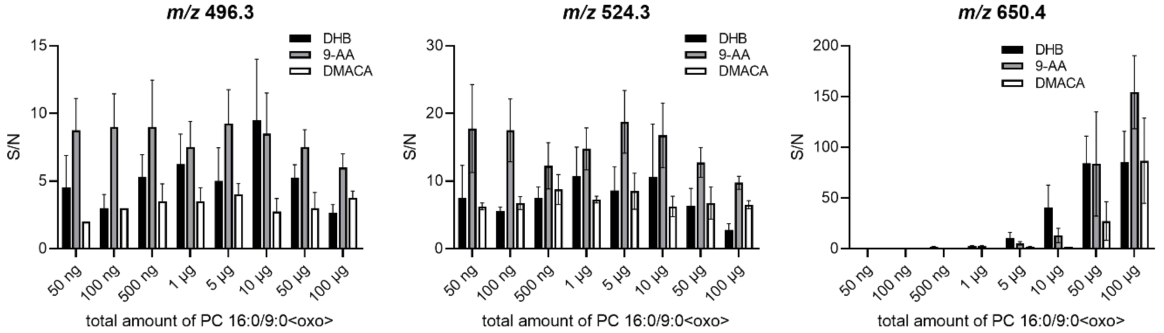


**Supporting Figure S2.** Signal-to-noise ratios of the proton adducts of LPC 16:0 (*m/z* 496.3) and LPC 18:0 (*m/z* 524.3) and the short-chain oxidized PC 16:0/9:0<oxo> (*m/z* 650.4) with increasing PC 16:0/9:0<oxo> amounts using the MALDI matrices 2,5-dihydroxybenzoic acid (DHB), 9-aminoacridine (9-AA) and 4-(dimethylamino)cinnamic acid (DMACA). Liver samples were spiked with different amounts of PC 16:0/9:0<oxo> and lipids were extracted as described in the Experimental Section. MALDI spectra were recorded in the positive ion mode. Data are shown as mean ± SD of four to six measurements from two liver samples (two to three measurements per sample).

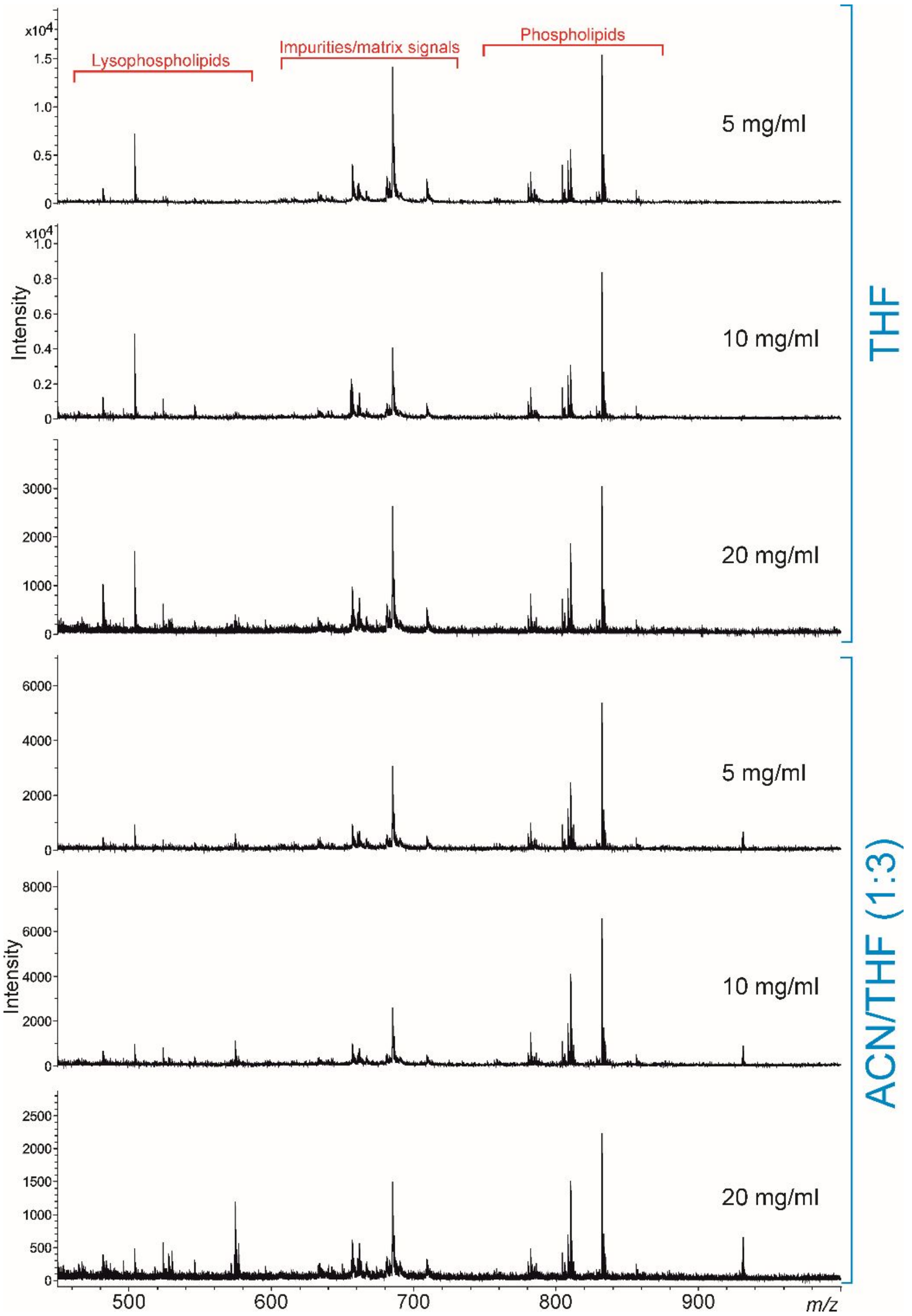


**Supporting Figure S3.** Performance of DMACA as MALDI matrix with organic rat liver extracts as selected examples. DMACA was mixed with either tetrahydrofuran (THF) or acetonitrile/THF (1:3, *v/v*) to get clear solutions of 5, 10 and 20 mg/ml DMACA. All matrix solutions were sonicated for 10 min. DMACA matrix solutions were mixed 1:40 with organic extracts from rat liver. MALDI mass spectra were recorded in the positive ion mode.

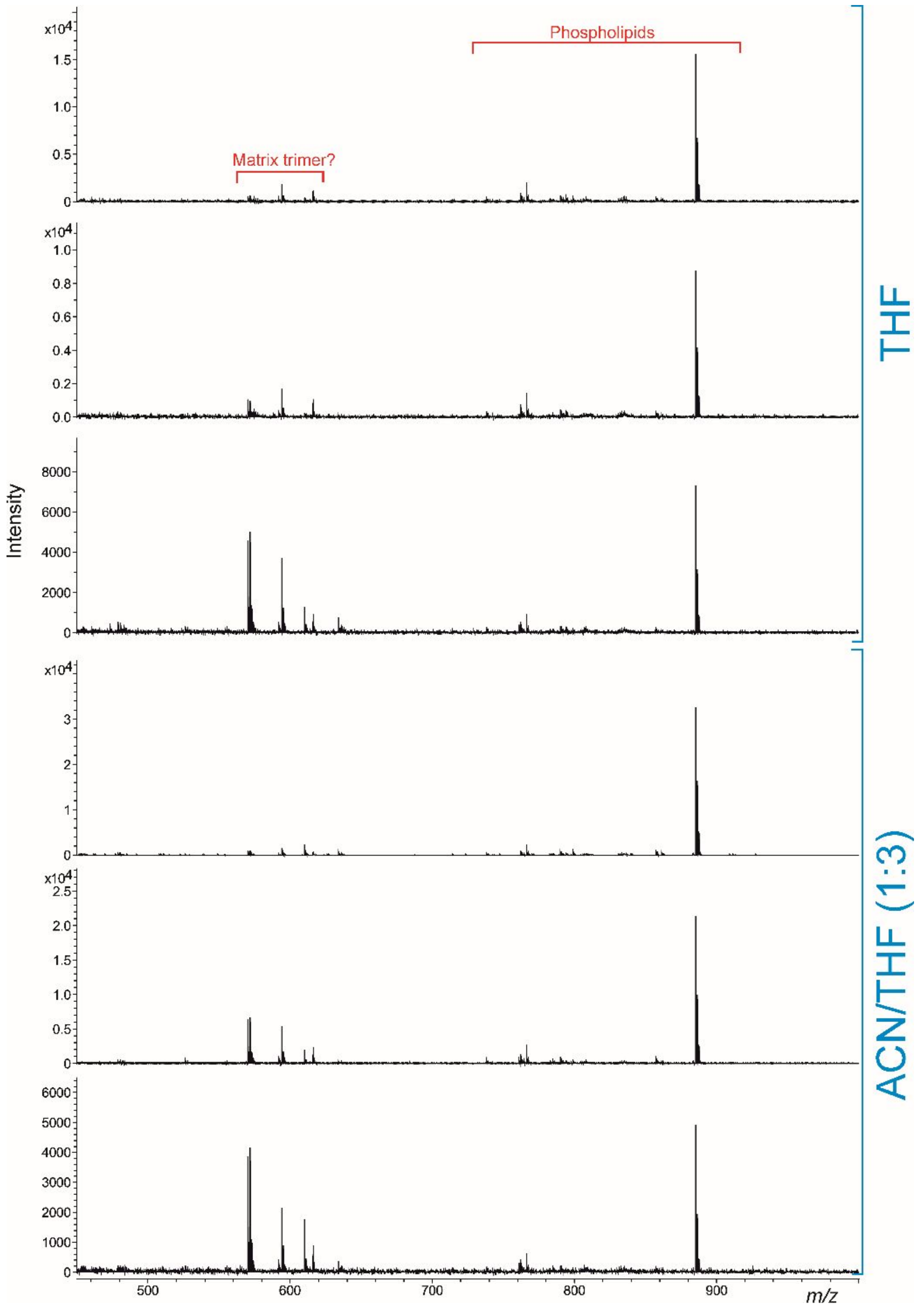


**Supporting Figure S4.** Performance of DMACA as MALDI matrix for the analysis of organic rat liver extracts. DMACA was mixed with either tetrahydrofuran (THF) or acetonitrile/THF (1:3, *v/v*) to get clear solutions of 5, 10 and 20 mg/ml DMACA. All matrix solutions were sonicated for 10 min. DMACA matrix solutions were mixed 1:40 with organic extracts from rat liver. MALDI mass spectra were recorded in the negative ion mode.

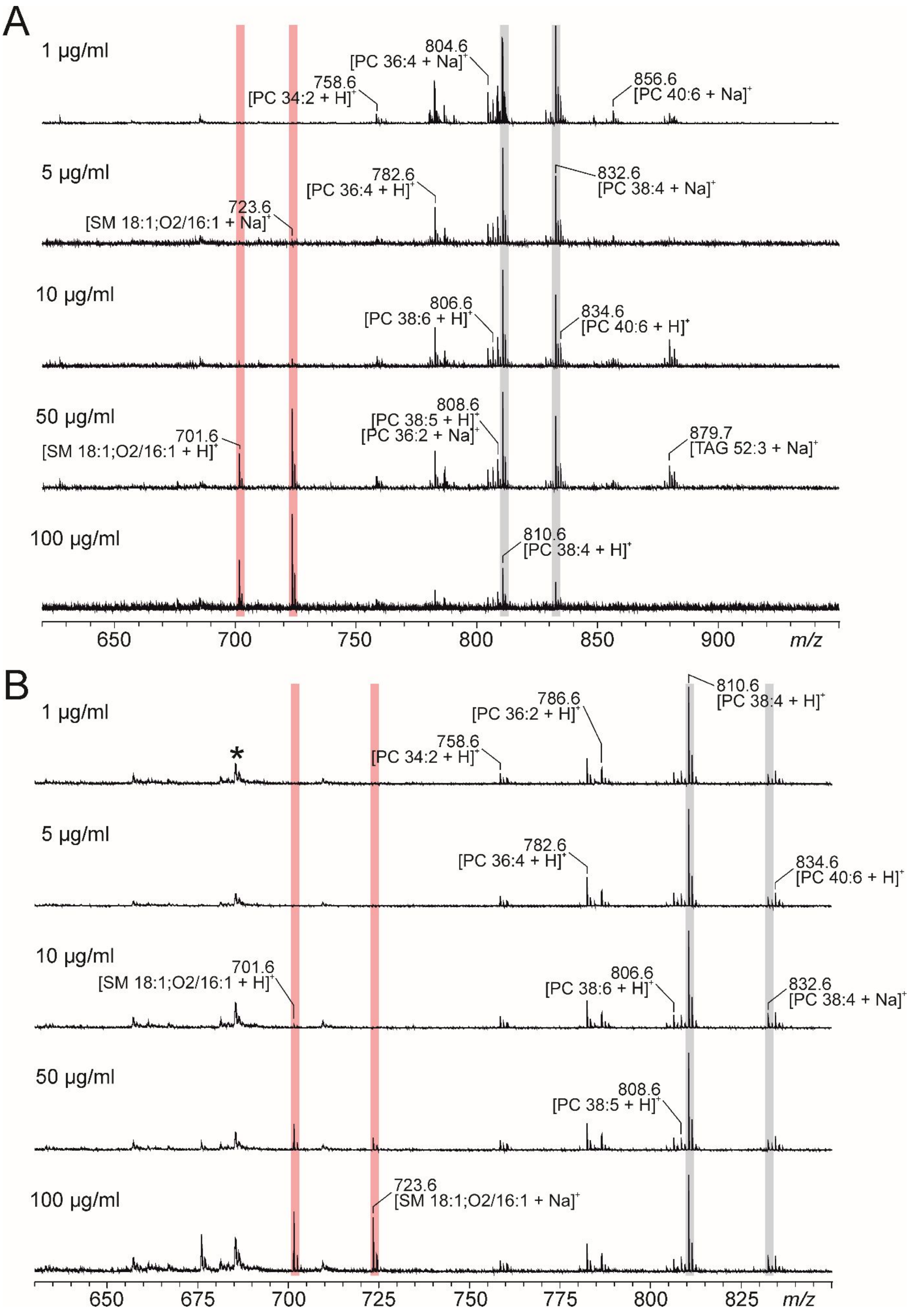
A
1 µg/ml
804.6
[PC 36:4 + Na]+
758.6
[PC 34:2 + H]+
856.6
[PC 40:6 + Na]+
5 µg/ml
782.6
[PC 36:4 + H]+
832.6
[PC 38:4 + Na]+
723.6
[SM 18:1;O2/16:1 + Na]+
10 µg/ml
806.6
[PC 38:6 + H]+
834.6
[PC 40:6 + H]+
50 µg/ml
808.6
[PC 38:5 + H]+
[PC 36:2 + Na]+
701.6
[SM 18:1;O2/16:1 + H]+
879.7
[TAG 52:3 + Na]+
100 µg/ml
810.6
[PC 38:4 + H]+
650
700
750
800
850
900
m/z
B
1 µg/ml
*
810.6
[PC 38:4 + H]+
786.6
[PC 36:2 + H]+
758.6
[PC 34:2 + H]+
5 µg/ml
782.6
[PC 36:4 + H]+
834.6
[PC 40:6 + H]+
10 µg/ml
701.6
[SM 18:1;O2/16:1 + H]+
806.6
[PC 38:6 + H]+
832.6
[PC 38:4 + Na]+
50 µg/ml
808.6
[PC 38:5 + H]+
100 µg/ml
723.6
[SM 18:1;O2/16:1 + Na]+
650
675
700
725
750
775
800
825
m/z

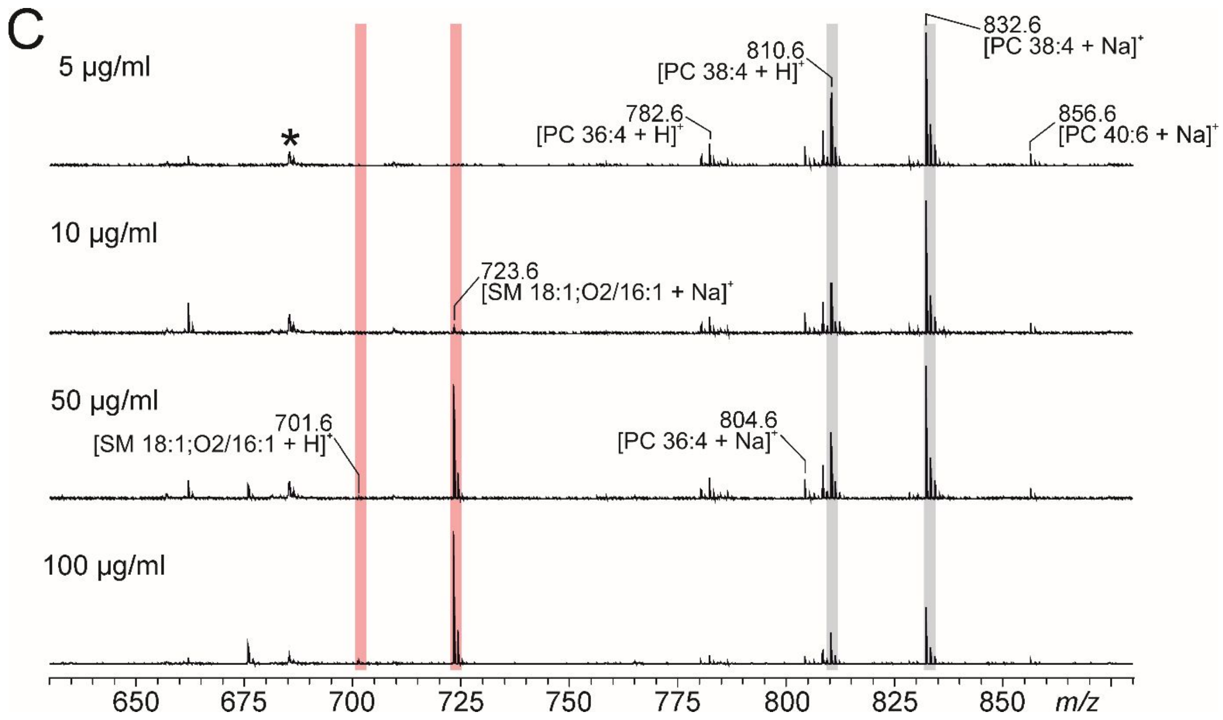


**Supporting Figure S5.** Positive ion MALDI-TOF mass spectra of rat liver extracts with different amounts of artificially added SM 18:1;O2/16:1. Different compounds were used as MALDI matrices and mixed with the organic liver extract: (A) 2,5-dihydroxybenzoic acid (0.5 M in methanol), mixed 1:1, (B) 9-aminoacridine (10 mg/ml in acetonitrile/2-propanol, 4:6, *v/v*), mixed 1:30, (C) 4-(dimethylamino)cinnamic acid (5 mg/ml in acetonitrile/THF, 1:3, *v/v*), mixed 1:40 with the organic extract. SM 18:1;O2/16:1 was mixed with rat liver extracts to get different concentrations of SM 18:1;O2/16:1 highlighted in pale red. The signals of PC 38:4, the most abundant phospholipid in rat liver, are highlighted in grey.

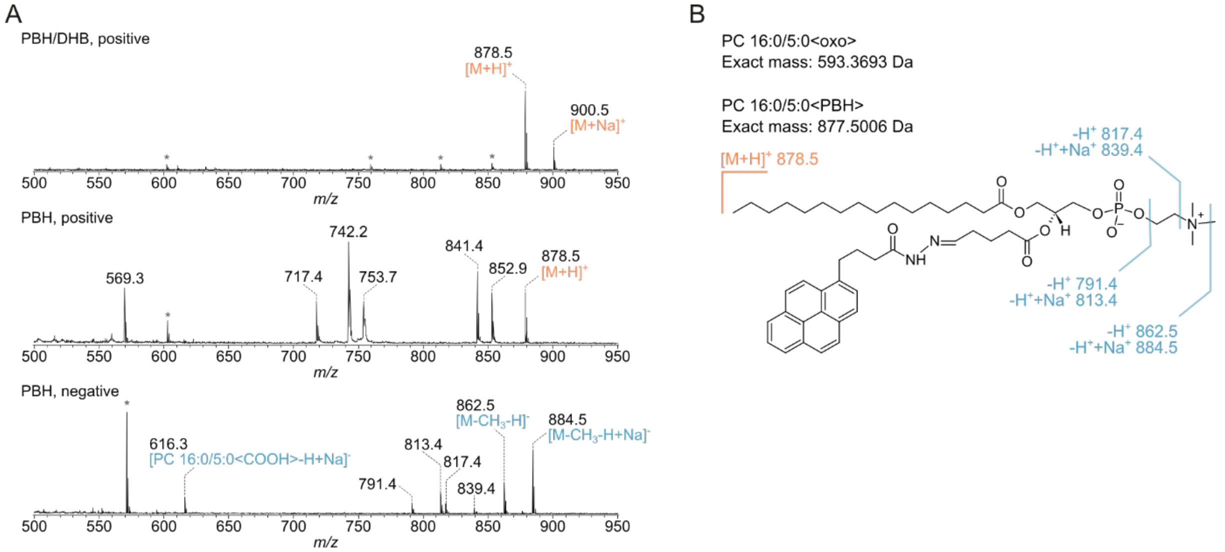


**Supporting Figure S6.** Positive and negative ion MALDI TOF mass spectra of derivatized PC 16:0/5:0<PBH> (A). Upper panel, PBH and DHB measured in the positive ion mode. Lower panel, only PBH measured in the negative ion mode. Peaks originating from the matrix are marked with an asterisk. Since DHB only provides intense matrix signals in the negative ion mode, this mode is not shown. Common peaks and general fragmentation patterns of PC 16:0/5:0<PBH> in MALDI MS (B).
